# Change in Age at Diagnosis of Oropharyngeal Cancer in the United States, 1975–2016

**DOI:** 10.3390/cancers12113191

**Published:** 2020-10-30

**Authors:** Brittany J. Cline, Matthew C. Simpson, Matthew Gropler, Aleksandr R. Bukatko, Eric Adjei Boakye, Kahee A. Mohammed, Nosayaba Osazuwa-Peters

**Affiliations:** 1 Saint Louis University School of Medicine, St. Louis, MO 63104, USA; brittany.cline@health.slu.edu; 2 Department of Otolaryngology-Head and Neck Surgery, Saint Louis University School of Medicine, St. Louis, MO 63104, USA; matthew.simpson@health.slu.edu (M.C.S.); matthew.gropler@childrenscolorado.org (M.G.); aleksandr.r.bukatko@health.slu.edu (A.R.B.); 3 Saint Louis University Cancer Center, St. Louis, MO 63110, USA; 4 Department of Population Science and Policy, Southern Illinois University School of Medicine, Springfield, IL 62702, USA; eadjeiboakye49@siumed.edu; 5 Department of Medicine, Washington University in St. Louis School of Medicine, St. Louis, MO 63104, USA; kaheem@wustl.edu; 6 Department of Head and Neck Surgery & Communication Sciences, Duke University School of Medicine, Durham, NC 27710, USA

**Keywords:** cancer surveillance, head and neck cancer (HNC), oropharyngeal cancer, age at diagnosis, Surveillance, Epidemiology, and End Results (SEER)

## Abstract

**Simple Summary:**

While previous studies have suggested that HPV-associated head and neck cancer (HNC) is mostly in the younger population, we found that in the last 40 years, the mean age at diagnosis of oropharyngeal cancer has been 60.3 years. We found that after an initially decrease leading up to the early 2000s, there has been a steady increase in age at diagnosis of oropharyngeal cancer since 2002. For non-oropharyngeal HNC, mean age at diagnosis has steadily increased throughout the last four decades. Finally, we found that age at diagnosis of HNC is higher among females in general, and lowest among blacks.

**Abstract:**

The emergence of the human papillomavirus (HPV) as the primary etiology of oropharyngeal cancer has changed head and neck cancer (HNC) epidemiology. This study described change in the age at diagnosis of oropharyngeal and non-oropharyngeal HNC in the United States in the last four decades. Using a retrospective cohort analysis, the Surveillance, Epidemiology, and End Results dataset from 1975 to 2016 was queried for eligible adult cases of HNC, grouped as oropharyngeal (*n* = 31,702) versus non-oropharyngeal (*n* = 87,108). Age at diagnosis was compared by gender (female, male) using independent t-test, and by race/ethnicity (Hispanic, non-Hispanic black, non-Hispanic white, non-Hispanic other) using analysis of variance. Joinpoint regression estimated yearly increases/decreases in age of diagnosis by sex and race/ethnicity through annual percent changes (APC), which were summarized with average annual percent changes (AAPC). Mean age at diagnosis for oropharyngeal cancer was 60.3 years. While there was initially a decrease in age at diagnosis, a 0.37% annual increase occurred from 2002 to 2016 (APC = 0.37, 95% confidence interval (CI) 0.28, 0.45). For non-oropharyngeal cancer, mean age at diagnosis was 63.2 years, with a continuous increase in age at diagnosis throughout the study period (1975–2016 AAPC = 0.08, 95% CI 0.04, 0.12). Females had higher average age at diagnosis than males for both sites, while blacks (57.4 years for oropharyngeal cancer; 59.0 years for non-oropharyngeal) had the lowest age at diagnosis of all races/ethnicity. Age at diagnosis of oropharyngeal cancer has increased significantly since 2002, while non-oropharyngeal HNC has increased significantly in the last four decades.

## 1. Introduction

The epidemiology of head and neck cancer (HNC) has evolved over time, mostly due to the decrease in smoking rates, and the concomitant emergence of the human papillomavirus (HPV) as the most common etiology of oropharyngeal cancer [1] HPV is linked to 70–90% of oropharyngeal cancer cases [2] and HPV-associated oropharyngeal cancer has increased in incidence in the United States between 54–225% in the last three decades [2,3,4,5]. Additionally, HPV-associated oropharyngeal cancer has now surpassed cervical cancer as the leading HPV-associated cancer in the United States, and this number is expected to continue to grow [6,7,8].

This changing epidemiology of HNC has also been associated with a change in the age at diagnosis, with several studies describing a shift from age of diagnosis in the sixth or seventh decade of life to an increasing incidence of HNC diagnosed in patients younger than 60 years, both in oropharyngeal and non-oropharyngeal cases [9,10,11,12,13,14,15,16,17]. However, more recent studies have indicated that average age of diagnosis for oropharyngeal cancer may not be as young as previously noted, and that the current and projected future increase in incidence of oropharyngeal cancer will be driven by the individuals 65 years and older, not younger [18,19,20,21,22]. 

Based on the above [9,10,11,12,13,14,15,16,17,18,19,20,21,22], it is evident that there have been changes in the age at diagnosis of HNC over time in the United States, both in oropharyngeal and non-oropharyngeal cases. The current study builds on recent studies [18,19,20,21,22], uses population-based cancer data, and tests the hypothesis that change in age at diagnosis of HNC in the United States in the last four decades differs based on race and gender as well oropharyngeal cancer status.

## 2. Results

### 2.1. Patient Characteristics

There were 31,702 first primary oropharyngeal cancer patients and 87,108 non-oropharyngeal (other) HNC patients diagnosed from 1975 to 2016 included in the study. Most patients were male (oropharyngeal cancer = 77%, other HNC = 74%) and non-Hispanic white (oropharyngeal cancer = 80%, other HNC = 79%). The average age at diagnosis over the entire study period was significantly lower for oropharyngeal cancer (60.3 years) compared with other HNC (63.2 years) (*p* < 0.01). Females had a significantly higher average age at diagnosis than males for oropharyngeal cancer (62.4 years versus 59.7 years) and other HNC (64.8 years versus 62.7 years) (*p* < 0.01). For oropharyngeal cancer, non-Hispanic Asian/Pacific Islander/American Indian/Alaska Native (API/AIAN) patients had the highest average age at diagnosis (61.8 years), followed by non-Hispanic whites (60.8 years), Hispanics (59.3 years), and non-Hispanic blacks (57.4 years) (ANOVA *p* < 0.01, pairwise comparisons *p* < 0.05). For other HNC, non-Hispanic whites had the highest average age at diagnosis (64.0 years), followed by Hispanics (61.8 years), non-Hispanic API/AIAN (60.6 years), and non-Hispanic blacks (59.0 years) (ANOVA *p* < 0.01, pairwise comparisons *p* < 0.05) (Table 1).

### 2.2. Overall Trends by Site

**Figure 1:**
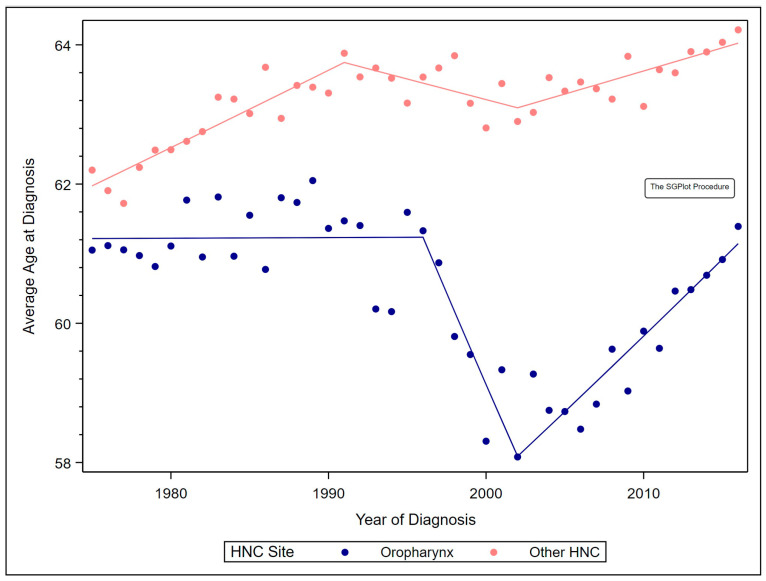
Observed average age at diagnosis by year and Joinpoint regression models by site.

For first primary oropharyngeal cancer, average age at diagnosis remained stable from 1975 to 1996, decreased 0.88% annually from 1996 to 2002 (annual percent changes (APC) = −0.88, 95% confidence interval (CI) −1.43, −0.32), and increased 0.37% annually from 2002 to 2016 (APC = 0.37, 95% CI 0.28, 0.45). These differing trends negated each other, resulting in a stable annual percent change (AAPC) from 1975 to 2016. For other HNC, average age at diagnosis increased 0.18% annually from 1975 to 1991 (APC = 0.18, 95% CI 0.13, 0.22), remained stable from 1991 to 2002, and increased 0.10% annually from 2002 to 2016 (APC = 0.10, 95% CI 0.04, 0.17). (Figure 1).

These trends resulted in an AAPC of 0.08% over the study period (AAPC = 0.08, 95% CI 0.04, 0.12). The average age at diagnosis of other HNC was 64.2 years in 2016, versus 62.2 years in 1975 (Table 2).

### 2.3. Trends by Site and Sex

**Figure 2:**
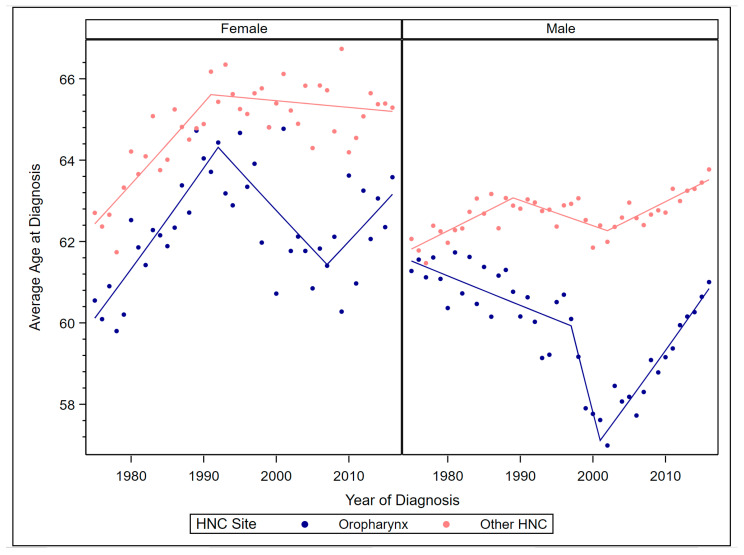
Observed average age at diagnosis by year and Joinpoint models by sex and site.

Among males and females, the average age at oropharyngeal cancer diagnosis was lower than at other HNC diagnosis (contrast *p* < 0.01) (Figure 2). 

For females, the average age at oropharyngeal cancer diagnosis increased 0.40% annually from 1975 to 1992 (APC = 0.40, 95% CI 0.25, 0.54), decreased 0.31% annually from 1992 to 2007 (APC = −0.31, 95% CI −0.53, −0.08), and remained stable from 2007 to 2016. This resulted in a stable AAPC from 1975 to 2016. The average age at other HNC diagnosis among females increased 0.31% annually from 1975 to 1991 (APC = 0.31, 95% CI 0.21, 0.41) but remained stable from 1991 to 2016, resulting in a 0.11% AAPC from 1975 to 2016 (AAPC = 0.11, 95% CI 0.06, 0.15). Among males, the average age at diagnosis of oropharyngeal cancer decreased by 0.12% annually from 1975 to 1997 (APC = −0.12, 95% CI −0.18, −0.06) and by 1.20% annually from 1997to 2001 (APC = −1.20, 95% CI −2.36, −0.02), but increased 0.42% annually from 2001 to 2016 (APC = 0.42, 95% CI 0.35, 0.49). This resulted in a stable AAPC from 1975 to 2016. For other HNC, average age at diagnosis among males increased 0.14% annually from 1975 to 1989 (APC = 0.14, 95% CI 0.09, 0.20), decreased 0.10% annually from 1989 to 2002 (APC = −0.10, 95% CI −0.18, −0.02), and increased 0.14% annually from 2002 to 2016 (APC = 0.14, 95% CI 0.08, 0.21). The AAPC in average age at other HNC diagnosis was 0.07% (AAPC = 0.07, 95% CI 0.03, 0.10). 

### 2.4. Trends by Site and Race/Ethnicity

**Figure 3:**
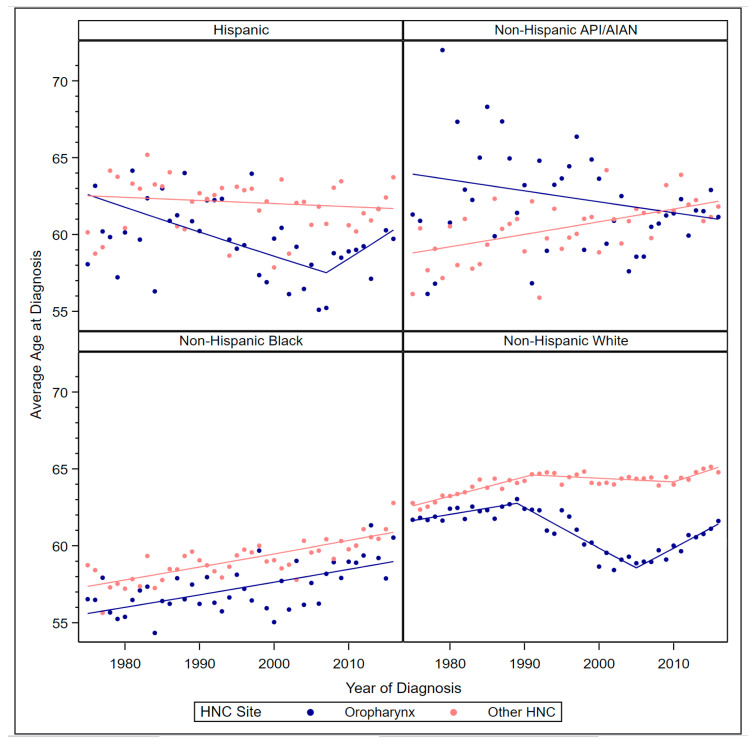
Observed average age at diagnosis by year and Joinpoint regression models by race/ethnicity and HNC site.

Among Hispanics, non-Hispanic blacks, and non-Hispanic whites, the average age at oropharyngeal cancer diagnosis was significantly lower than at other HNC diagnosis (contrast *p* < 0.01), but among non-Hispanic API/AIAN it was significantly higher than at other HNC diagnosis (contrast *p* < 0.01) (Figure 3). Average age for oropharyngeal cancer diagnosis among Hispanics decreased 0.26% annually from 1975 to 2007 (APC = −0.26, 95% CI −0.42, −0.11) but increased 0.53% annually from 2007 to 2016 (APC = 0.53, 95% CI 0.01, 1.04), resulting in a stable AAPC from 1975 to 2016. The average age at other HNC diagnosis among Hispanics also remained stable from 1975 to 2016. Among non-Hispanic API/AIAN, average age at diagnosis of oropharyngeal cancer decreased 0.11% annually from 1975 to 2016 (APC = −0.11, 95% CI −0.23, −0.0008), but average age at other HNC diagnosis increased 0.14% annually from 1975 to 2016 (APC = 0.14, 95% CI 0.07, 0.20). Among non-Hispanic blacks, average age at diagnosis increased 0.14% annually for both oropharyngeal cancer (APC = 0.14, 95% CI 0.09, 0.20) and other HNC (APC = 0.14, 95% CI 0.11, 0.18). Among non-Hispanic whites, average age at oropharyngeal cancer diagnosis increased 0.13% annually from 1975 to 1989 (APC = 0.13, 95% CI 0.02, 0.24), decreased 0.43% annually from 1989 to 2005 (APC = −0.43, 95% CI 0000.53, −0.33), and increased 0.43% annually from 2005 to 2016 (APC = 0.43, 95% CI 0.32, 0.54). These differing trends resulted in a stable AAPC from 1975 to 2016.

For other HNC, average age at diagnosis among non-Hispanic whites increased 0.20% annually from 1975 to 1991 (APC = 0.20, 95% CI 0.15, 0.24), remained stable from 1991 to 2010, and increased 0.25% annually from 2010 to 2016 (APC = 0.25, 95% CI 0.01, 0.48), resulting in an AAPC of 0.10% from 1975 to 2016 (APC = 0.10, 95% CI 0.05, 0.14). 

## 3. Discussion

This study aimed to profile the changes in age of diagnosis of oropharyngeal and non-oropharyngeal HNC in the United States, building on previous studies [18,19,20,21,22] to assess age-based trends in the last four decades, stratified by race and gender. Analysis was based on squamous cell carcinoma of the head and neck. We found that average age of diagnosis of oropharyngeal cancer has varied since 1975, dropping significantly low and then rising gradually back to the mean age at study onset. Conversely, for other non-oropharyngeal HNC, we found that the average age at diagnosis increased over the last 40 years. This overall pattern we found differed based on race. For example, we saw that for both oropharyngeal cancer and other HNC, the age at diagnosis among blacks has steadily increased in the last 40 years. If the epidemiology of HNC continue to evolve as predicted due to oropharyngeal cancer as well as primary prevention of oral HPV through the HPV vaccine [21,23] it will be interesting to see race-based changes in incidence and age at diagnosis of HNC in the next few decades.

While oropharyngeal cancer had been repeatedly characterized in the literature as the HPV-driven epidemic of the young, several recent studies have maintained that this perceived drop in age of diagnosis is due to the age–birth cohort effect [17,18,19,20,21]. In particular, Tota et al. described an era that coincided with the emergence of the HIV/AIDS crisis in the United States, and that the age–birth cohort in the early and late 1980s with HPV-associated oropharyngeal cancer were largely responsible for the drop in age at diagnosis, suggesting a younger age profile for patients with oropharyngeal cancer [21]. As that cohort has aged over the last two decades, there are indications that the age at diagnosis has increased significantly [21]. On the basis of a 40-year analysis, we agree with the most recent understanding [18,19,20,21] and conclude that oropharyngeal cancer is not a disease of young people, as we estimated a mean age at diagnosis of over 60 years in the course of the last four decades. Additionally, the sharp increase in age at diagnosis we saw since around 2002 and the steady climb until 2016 (the cut-off year for our data) supports Tota et al.’s projection that age at diagnosis that might drive incidence of oropharyngeal cancer in the next decade might be 65 years and older [21]. The sharp increase in age at diagnosis we saw in our trend analysis as well as Tota et al.’s projections [21] both have therapeutic and population health implications, from prevention to treatment to survival of HPV-associated oropharyngeal cancer.

In contrast to the fluctuation in age at diagnosis of oropharyngeal cancer, we found a slow but steady increase in age at diagnosis for the non-oropharyngeal HNC sites over the last 40 years, and by 2016, mean age at diagnosis was 64 years, a 0.08% annual increase since 1975. In the context of oropharyngeal cancer, we conclude that the age at diagnosis for non-oropharyngeal HNC patients remains significantly older than for patients with oropharyngeal cancer. Age is an important factor in HNC treatment decisions and outcomes [24] and sociodemographic factors prognostic in survival differ among elderly versus nonelderly patients of HNC [25]. If the trend we observed over 40 years continues to hold, along with the projected aging of the both general and the cancer survivor population of the United States [26,27], the role of the patient’s age and its associated physical and cognitive factors in HNC cancer care and therapeutic options might become even more important in the future [25].

The patterns seen in age at diagnosis for both oropharyngeal and non-oropharyngeal HNC differed based on race and gender. We found that while age at diagnosis among blacks continued to increase throughout the study period for both oropharyngeal and non-oropharyngeal cases, their age at diagnosis was lower than that of whites for both oropharyngeal (57 versus 61 years) and non-oropharyngeal HNC cases (61 versus 65 years). It is unclear why these patterns exist; however, it is interesting in the context of disparities associated with mortality. For example, black HNC patients, while presenting at a younger age, have worse life expectancy, and worse overall and disease-specific mortality than whites [28,29]. In fact, HNC has the third worst black versus white mortality differences of all cancers in the United States [30]. We also report a steady increase in age at diagnosis for non-oropharyngeal head and neck cancer patients who are API/AIAN, while there was a decrease in age of presentation among those with oropharynx disease. Very few studies have described the epidemiology of head and neck cancer among API/AIAN patients, and these studies have mostly focused on stage of presentation and survival/mortality [31,32,33,34]. These studies have consistently reported worse head and neck cancer outcomes in this minority population, and our study adds to the existing literature by describing age-based trends in this population in the last four decades.

### 3.1. Clinical and Public Health Implications

A recent study aptly described elderly oropharyngeal cancer patients as the “new face of the HPV epidemic” [35]. As shown in the current study and other previous studies, the shift in average age of presentation of oropharyngeal cancer from 50s to mid-60s and older has several implications, including concerns related to enrollment in clinical trials [36], and effectiveness and safety of de-escalation of treatment regimens [37]. Elderly patients are less likely to be involved in clinical trials and new therapies [25,36,38,39,40] and practitioners might be more concerned about the potential benefits versus harms associated with the complex, extensive procedures required especially for multimodal care in advanced-stage disease [24,36,40,41]. Additionally, the oft-referenced clinical picture of an oropharyngeal cancer patients as an otherwise healthy individual with low comorbidity burden is severely challenged by the known burden of comorbid conditions naturally associated with the process of aging [21,42]. With oropharyngeal cancer projected to be the third most common cancer among 55–69 year-old white, non-Hispanic males by 2045 [43], managing elderly HNC might become the norm rather than the exception in decades to come. It is therefore critical that focus begins to shift towards developing new treatment paradigms and interventions that offer optimal care for the elderly patients with HNC [35]. If immunotherapy proves to be more tolerable among the elderly without compromising efficacy of treatment, this might be an important treatment option that might be tailored based on the clinical profile of elderly HNC patients in the future [44].

From a public health and prevention perspective, the increasing age at diagnosis of HNC found in this study is important. For non-oropharyngeal cancer, which is mostly associated with tobacco use, it is important that public health messaging about primary prevention through tobacco cessation is sustained [45]. Additionally, while the United States Preventative Task Force have found no mortality benefit in mass asymptomatic screenings in primary care settings [46], some researchers and expert groups have suggested that providing oral cavity screenings for early detection and education about oral cancer risk factors remain beneficial especially for high-risk groups [47,48,49,50,51]. For oropharyngeal cancer, primary cancer screenings may not be effective; however, individuals might benefit from HPV vaccinations, which have been shown to prevent oral HPV 16 and 18, which cause over 90% of all HPV-positive oropharyngeal cancer [23]. The need for prevention of HPV-associated oropharyngeal cancer could not be more emphasized by the fact that it has now surpassed cervical cancer as the leading HPV-associated cancer in the United States [6]. In addition, unlike cervical cancer, oropharyngeal cancer is predominated by males, and future increase in incidence is projected to be driven by elderly males [21,52]. The recent approval of the HPV vaccine for individuals up to 45 years of age might yet impact prevalence of oral HPV in the future since it might take decades from HPV infection to malignancy [53]. Along the same lines, the June 12, 2020 announcement by the Food and Drug Administration expanding the indications for the HPV vaccine to include prevention of HPV-associated HNC would likely lead to more clinical providers discussing the HPV vaccine among older adults 45 years of age or less who are now eligible for the vaccine even after being sexually active [54]. 

### 3.2. Strengths and Limitations

Our study has some limitations. First, this was a descriptive, retrospective study and so we cannot draw causal inferences. Second, our analysis was limited to data in the Surveillance, Epidemiology, and End Results (SEER) database, which only covers a fraction of the United States population. Third, the SEER data used for this study does not have HPV positive tumor status for the oropharyngeal cancer cases we analyzed; therefore, any suggested HPV-association was by anatomical site as proxy.

Notwithstanding these limitations, this study makes an important contribution to the HNC and cancer surveillance literature by providing a comprehensive description of changing patterns of age at diagnosis of HNC, both oropharyngeal and non-oropharyngeal disease, based on race and gender. The strength of the study includes providing age at diagnosis from a premium quality, population-based cancer registry. It builds on Tota et al.’s age-based trends study [21] to describe the changes in age at diagnosis of HNC over a four-decade span. The implications of the study are important to population health as well as clinically, as more individuals outlive their primary HNC diagnosis and grow older as cancer survivors.

## 4. Methods

### 4.1. Data Source

This study included patients from the Surveillance, Epidemiology, and End Results (SEER) 9 database from the National Cancer Institute [55]. SEER 9 includes cancer patients from registries in 9 areas of the United States, covering about 9.4% of the population from 1975 to 2016 [56]. SEER’s nationally representative data and quality improvement practices ensure that the database can provide insight into cancer trends in the United States [57]. On October 1, 2019, the Saint Louis University Institutional Review Board (IRB) approved that no further human subject review was needed as the SEER data is publicly available and de-identified.

### 4.2. Study Population

This study included patients diagnosed with first primary malignant head and neck squamous cell carcinoma from 1975 to 2016. This was defined based on the International Classification of Diseases—Oncology, third edition (ICD-O-3), as any malignancy of the oral cavity/pharynx primary sites C00.0–C14.8, nose/nasal cavity (C30.0, C31.0–C31.9), and larynx (C32.0–C32.9) with ICD-O-3 squamous cell histologic type (8050–8084 or 8120–8131). Malignancies at these sites were dichotomized into oropharyngeal cancer (ICD-O-3 primary sites C1.9, C2.4, C2.8, C5.1–C5.2, C9.0–C10.9, C14.0–C14.8) and other HNC for this study. (See Table S1 and Figure S1 for the results for individual anatomic sites).

### 4.3. Data Analysis

We used SEER*Stat version 8.3.5 (Surveillance Research Program, National Cancer Institute, Bethesda, MD, USA) to retrieve patient-level information. SAS version 9.4 (SAS Institute, Cary, NC, USA) calculated differences in average age at diagnosis of oropharyngeal squamous cell carcinoma (OPSCC) and other HNC by sex (female, male) using independent samples t-tests and race/ethnicity (Hispanic, non-Hispanic API/AIAN, non-Hispanic black, non-Hispanic white) using analysis of variance (ANOVA) with Bonferroni adjustments for pairwise comparisons. ANOVA with contrasts estimated differences in average age at diagnosis between sites within each demographic group.

Joinpoint Regression Program version 4.7.0.0 (Statistical Methodology and Applications Branch, Surveillance Research Program, National Cancer Institute, Bethesda, MD, USA) determined the time periods for significant increases or decreases in annual average age at first primary OPSCC/other HNC diagnosis through Joinpoint regression models. These models determined the starting and ending years of average age increases/decreases (Joinpoints) and then estimated the annual percentage change (APC) and 95% confidence intervals (CI) based on a regression model between the two Joinpoint years. The final Joinpoint models were based on log-transformed annual average ages to better ensure normality of residuals [58]. The permutation test method determined the model with the fewest number of Joinpoints necessary to effectively characterize trends with a maximum of 5 Joinpoints [59]. Trends with multiple APCs were summarized with average APCs (AAPC) covering the entire 1975–2016 time range. We computed Joinpoint regressions for OPSCC and other HNC stratified by sex and race. Significance for all tests was set at α = 0.05, and all tests were two-tailed.

## 5. Conclusions

There have been changes in age at diagnosis of HNC in the United States in the last four decades. While there was decrease in age at diagnosis of oropharyngeal cancer in the 1980s to early 2000s, we show an increasing age at diagnosis since 2002, which confirms that there may be more elderly oropharyngeal cancer patients in the United States in the future if the current pattern of increasing age at diagnosis persists.

## Figures and Tables

**Table 1:** Characteristics of head and neck cancer (HNC) patients, Surveillance, Epidemiology, and End Results (SEER) 9, 1975–2016.

Characteristics	Oropharyngeal Cancer			Other Head and Neck Cancer		
	*n* (%)	Mean Age at Diagnosis (SD)	*p*-Value	*n* (%)	Mean Age at Diagnosis (SD)	*p*-Value
Overall	31,702 (100)	60.3 (10.8)	-	87,108 (100)	63.2 (12.7)	-
Sex			<0.01			<0.01
Female	7245 (22.9)	62.4 (11.9)		22,618 (26.0)	64.8 (14.1)	
Male	24,457 (77.1)	59.7 (10.4)		64,490 (74.0)	62.7 (12.2)	
Race			<0.01			<0.01
Hispanic	1234 (3.9)	59.3 (11.0)		3378 (3.9)	61.8 (13.7)	
Non-Hispanic API/AIAN	1166 (3.7)	61.8 (12.6)		5556 (6.4)	60.6 (14.7)	
Non-Hispanic Black	3920 (12.4)	57.4 (10.4)		8718 (10.0)	59.0 (11.7)	
Non-Hispanic White	25,310 (79.8)	60.8 (10.7)		69,060 (79.3)	64.0 (12.5)	
Non-Hispanic Unknown	72 (0.2)	-		396 (0.5)	-	

Note: API/AIAN = Asian/Pacific Islander/American Indian/Alaska Native.

**Table 2:** Annual percentage changes and average annual percentage changes of average age at diagnosis from 1975 to 2016 by HNC site.

Characteristics	Oropharyngeal Cancer			Other Head and Neck Cancer		
	Year Range	APC (95% CI)	Average APC from 1975 to 2016 (95% CI)	Year Range	APC (95% CI)	Average APC from 1975 to 2016 (95% CI)
**Overall**			0.00 (−0.09, 0.09)			**0.08 (0.04, 0.12)**
	1975–1996	0.00 (−0.06, 0.07)		1975–1991	**0.18 (0.13, 0.22)**	
	1996–2002	**−0.88 (−1.43, −0.32)**		1991–2002	−0.09 (−0.19, 0.01)	
	2002–2016	**0.37 (0.28, 0.45)**		2002–2016	0.10 (0.04, 0.17)	
**Sex**						
Female			0.12 (−0.01, 0.25)			**0.11 (0.06, 0.15)**
	1975–1992	**0.40 (0.25, 0.54)**		1975–1991	**0.31 (0.21, 0.41)**	
	1992–2007	**−0.31 (−0.53, −0.08)**		1991–2016	−0.03 (−0.08, 0.03)	
	2007–2016	0.31 (−0.07, 0.69)				
Male			−0.03 (−0.15, 0.09)			**0.07 (0.03, 0.10)**
	1975–1997	**−0.12 (−0.18, −0.06)**		1975–1989	**0.14 (0.09, 0.20)**	
	1997–2001	**−1.20 (−2.36, −0.02)**		1989–2002	**−0.10 (−0.18, −0.02)**	
	2001–2016	**0.42 (0.35, 0.49)**		2002–2016	**0.14 (0.08, 0.21)**	
**Race/Ethnicity**						
Hispanic			−0.09 (−0.25, 0.07)			−0.03 (−0.10, 0.04)
	1975–2007	**−0.26 (−0.42, −0.11)**		1975–2016	−0.03 (−0.10, 0.04)	
	2007–2016	**0.53 (0.01, 1.04)**				
Non-Hispanic API/AIAN			**−0.11 (−0.23, −0.0008)**			**0.14 (0.07, 0.20)**
	1975–2016	**−0.11 (−0.23, −0.0008)**		1975–2016	**0.14 (0.07, 0.20)**	
Non-Hispanic Black			**0.14 (0.09, 0.20)**			**0.14 (0.11, 0.18)**
	1975–2016	**0.14 (0.09, 0.20)**		1975–2016	**0.14 (0.11, 0.18)**	
Non-Hispanic White			−0.01 (−0.07, 0.05)			**0.10 (0.05, 0.14)**
	1975–1989	**0.13 (0.02, 0.24)**		1975–1991	**0.20 (0.15, 0.24)**	
	1989–2005	**−0.43 (−0.53, −0.33)**		1991–2010	−0.04 (−0.08, 0.01)	
	2005–2016	**0.43 (0.32, 0.54)**		2010–2016	**0.25 (0.01, 0.48)**	

Note: API/AIAN = Asian/Pacific Islander/American Indian/Alaska Native; APC = Annual Percentage Change; CI = Confidence Interval.

## Supplementary Materials

The following are available online at https://www.mdpi.com/2072-6694/12/11/3191/s1, Figure S1: Observed average age at diagnosis by year and Joinpoint regression models by head and neck anatomic subsite, Table S1: Annual percentage changes and average annual percentage changes average age at diagnosis from 1975–2016 by head and neck anatomic subsites.
