# Supplementary material for: Change in Age at Diagnosis of Oropharyngeal Cancer in the United States, 1975–2016
Source: Cancers (Basel). 2020 Oct 30;12(11):3191. doi: 10.3390/cancers12113191 (PMC7693134; doi:10.3390/cancers12113191)
Supplement: Supplementary file 1 [file cancers-12-03191-s001.pdf]

cancers

MDPI

Article

Change in age at diagnosis of oropharyngeal cancer in the United States, 1975-2016

Brittany J Cline,, Matthew C Simpson, Matthew Gropler, Aleksandr R Bukatko, Eric Adjei Boakye, Kahee A Mohammed and Nosayaba Osazuwa-Peters

Supplementary Information

Table S1. Annual percentage changes and average annual percentage changes average age at diagnosis from 1975–2016 by head and neck anatomic subsites.

Characteristics	*n* (%)	Mean Age at Diagnosis (SD)	*p*-value	Year range	APC (95% CI)	Average APC from 1975–2016 (95% CI)
Site			**< 0.01**			
Hypopharynx	7044 (5.9)	63.2 (10.4)				0.01 (−0.03, 0.05)
				1975–2016	0.01 (−0.03, 0.05)	
Larynx	33768 (28.4)	63.2 (10.9)				0.12 (0.08, 0.16)
				1975–1985	0.36 (0.21, 0.52)	
				1985–2016	0.04 (0.01, 0.07)	
Nasopharynx	4163 (3.5)	54.4 (15.1)				0.07 (0.00, 0.15)
				1975–2016	0.07 (0.00, 0.15)	
Oral Cavity	39178 (33.0)	64.1 (13.8)				0.05 (0.03, 0.06)
				1975–2016	0.05 (0.03, 0.06)	
Oropharynx	31702 (26.7)	60.3 (10.8)				0.00 (−0.09, 0.09)
				1975–1996	0.00 (−0.06, 0.07)	
				1996–2002	−0.88 (−1.43, −0.32)	
				2002–2016	0.37 (0.28, 0.45)	
Sinonasal	2955 (2.5)	64.4 (13.5)				0.03 (−0.11, 0.18)
				1975–1991	0.36 (0.06, 0.67)	
				1991–2016	−0.18 (−0.33, −0.02)	

Note: API/AIAN = Asian/Pacific Islander/American Indian/Alaska Native; APC = Annual Percentage Change.

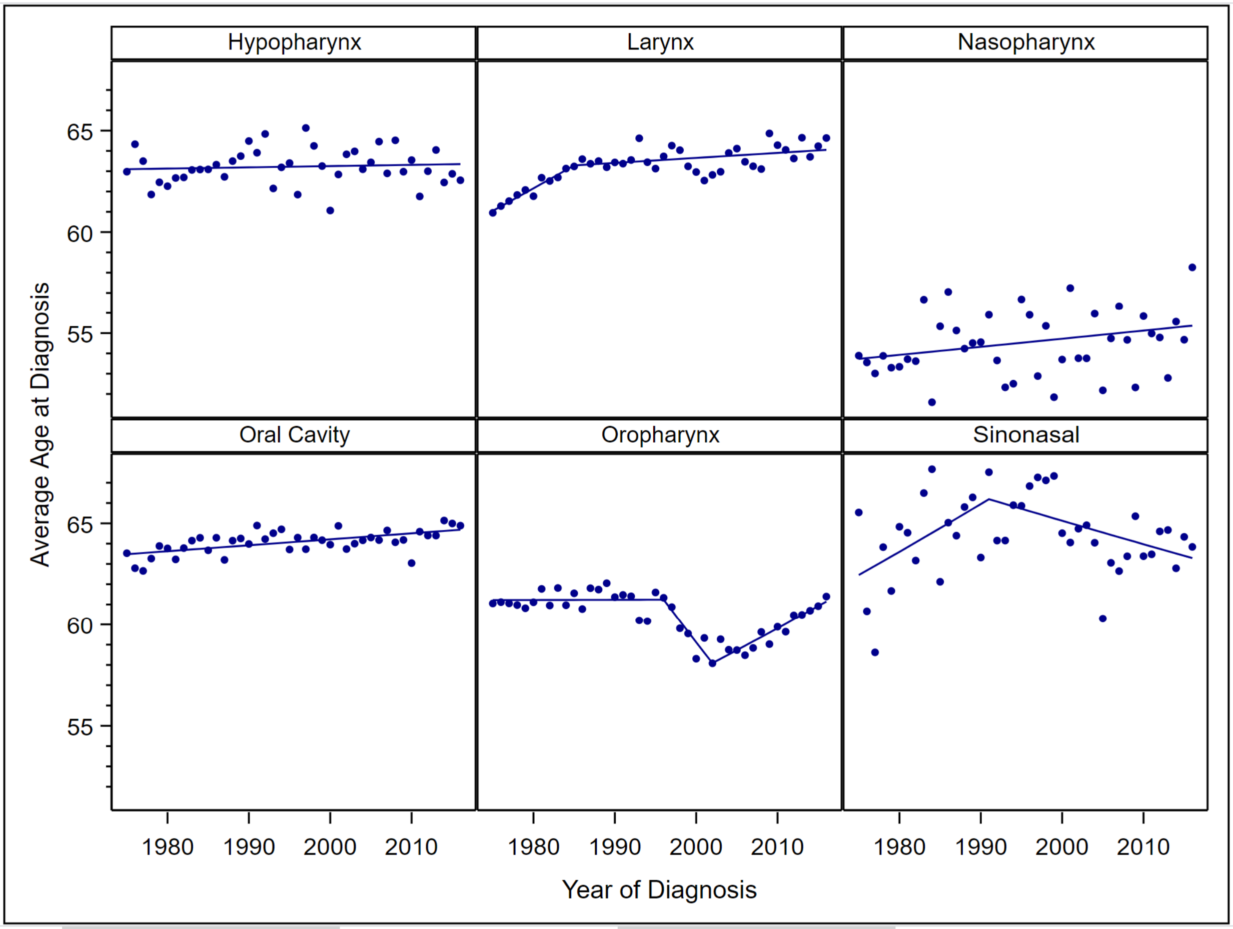

Figure S1. Observed average age at diagnosis by year and Joinpoint regression models by head and neck anatomic subsite.

Publisher's Note: MDPI stays neutral with regard to jurisdictional claims in published maps and institutional affiliations.

© 2020 by the authors. Licensee MDPI, Basel, Switzerland. This article is an open access article distributed under the terms and conditions of the Creative Commons Attribution (CC BY) license (http://creativecommons.org/licenses/by/4.0/).
